# Comparative effectiveness of single vs. combined exercise modalities on sleep disorders in Chinese adolescents: a systematic review and network meta-analysis

**DOI:** 10.3389/fpubh.2026.1865603

**Published:** 2026-07-09

**Authors:** Xiaoyan Li, Wei Gao, Zhiheng Li, Ji Zhu, Yanhao Wang, Ming Li

**Affiliations:** School of Physical Education and Sports Science, Fujian Normal University, Fuzhou, Fujian, China

**Keywords:** adolescents, China, combined exercise, network meta-analysis, single exercise, sleep disorders

## Abstract

**Objective:**

To systematically compare the effectiveness of single vs. combined exercise modalities for sleep disorders in Chinese adolescents and explore optimal exercise prescription variables using network meta-analysis.

**Methods:**

Eight databases were searched up to May 31, 2025. Stata 18.0 was used for analysis, with intervention rankings determined by SUCRA probabilities. The GRADE approach assessed evidence certainty.

**Results:**

Fourteen randomized controlled trials involving 1,375 participants were included. The combination of aerobic and mind-body exercise (AE + ME) showed the highest probability of being most effective (SUCRA = 87.1%), followed by mind-body exercise alone (ME, SUCRA = 71.3%). Pairwise comparisons confirmed that ME, AE, AE + RE, and AE + ME all significantly improved sleep disorders compared to controls. The optimal regimen consisted of 40–60 min sessions, ≥5 times per week [MD = 4.05, 95% CI (2.09, 6.01)], for less than 8 weeks [MD = 2.34, 95% CI (0.32, 4.37)]. GRADE assessment indicated moderate certainty for ME, aerobic exercise, and these optimal parameters.

**Conclusion:**

Mind-body exercise performed for 40–60 min per session [MD = 3.18, 95% CI (2.20, 4.15)], at least five times per week, for less than 8 weeks, may be the most effective approach for improving sleep disorders in Chinese adolescents, supported by moderate certainty evidence. Findings should be interpreted cautiously due to low or very low certainty for most combined modalities.

**Systematic review registration:**

CRD420251118367.

## 1 Introduction

Sleep disorders, a prevalent global health issue, are particularly common and treatable among adolescents (1). In China, teenagers face significant academic pressure from crucial examinations such as the high school and college entrance exams. This specific developmental stage, characterized by ongoing physical and psychological maturation, coupled with increased academic workloads and extensive use of electronic devices and social platforms (2, 3), severely compromises their sleep quality. Furthermore, accelerated lifestyles and heightened societal pressures have led to a rapid rise in sleep disorder rates among Chinese youth (4). Research indicates that short sleep duration and unhealthy sleep patterns are widespread among Chinese university students (5). Prominent sleep disturbances are closely linked to various mental health issues (6), including depression (7), anxiety (8), and psychotic symptoms (9). Moreover, studies confirm that sleep disorders contribute to a decline in immune function, elevated levels of depression and anxiety, endocrine imbalances, metabolic dysregulation, and increased risks for type 2 diabetes and cardiovascular diseases (10–12). This phenomenon is closely tied to the unique biopsychosocial transitions of adolescence. The activation of the hypothalamic-pituitary-gonadal axis delays melatonin secretion, causing a phase shift in circadian rhythms (13). Concurrently, academic stress and exposure to blue light from electronic devices further disrupt the sleep-wake cycle (14). Chronic sleep disorders not only impair cognitive function and academic performance but also significantly increase the risk of depression and metabolic syndrome (15).

Current treatments for sleep disorders include pharmacological and non-pharmacological approaches. However, clinical intervention faces a dual dilemma. First-line therapy such as Cognitive Behavioral Therapy for Insomnia is difficult to access and scale due to systemic barriers (16). Meanwhile, while benzodiazepines can provide short-term symptom relief, their long-term use in adolescents carries risks of tolerance and dependence (17). Moreover, prolonged pharmacological treatment can alter sleep architecture and increase the risk of depression, anxiety, and cognitive impairment (18). Therefore, there is an urgent need to prioritize non-pharmacological interventions for managing sleep disorders in adolescents. Exercise intervention has emerged as a highly promising solution due to its safety, accessibility, and multifaceted physiological regulatory effects. Mechanisms by which exercise improves sleep are well-documented in adult populations. Numerous studies indicate that exercise, as a complementary non-pharmacological intervention, positively impacts sleep in individuals with sleep disorders. Modalities such as Tai Chi (19), aerobic exercise (20), and resistance training (21) have all been shown to improve sleep to varying degrees. For instance, a study by Cui et al. (22), demonstrated that an 8-week regimen of the Qigong exercise performed 5 times per week for 60 min per session, significantly reduced the total Pittsburgh Sleep Quality Index (PSQI) score in the experimental group, indicating marked improvement in sleep quality. Similarly, Yuan et al. (23), found that various combined exercise interventions over 8 weeks significantly improved subjective sleep quality in adolescents with sleep disorders. Xu et al. (24), conducted a 12-week, 3-times-weekly circuit resistance training program, which significantly lowered total PSQI scores while also ameliorating negative psychological states such as anxiety and depression. However, adolescents possess unique characteristics—including hormonal fluctuations, heightened neuroplasticity, and a sensitive hypothalamic-pituitary-adrenal axis stress response—which may alter the dose-response relationship between exercise and sleep (25).

Although these findings are encouraging, current research on treating sleep disorders in Chinese adolescents still presents key limitations. First, existing studies often focus on single intervention modalities, with insufficient systematic comparisons of combined approaches. Second, given the rapid growth, development, and physiological distinctiveness of children and adolescents (26), future research urgently needs to explore the optimal duration, frequency, and implementation period of exercise interventions to formulate more personalized and precise therapeutic strategies for improving sleep quality in this population. To address these gaps, this study employs a network meta-analysis (NMA) to synthesize the evidence. Compared to traditional meta-analysis, NMA can integrate both direct and indirect comparison evidence, simultaneously ranking and evaluating the efficacy of multiple interventions (27).

This study aims to compare the effectiveness of single vs. combined exercise modalities and to determine which intervention period, session duration, and exercise frequency are most effective for improving sleep disorders in Chinese adolescents. The findings are intended to provide effective strategies for enhancing sleep quality in individuals with sleep disorders and offer important evidence for clinical practice.

## 2 Materials and methods

This study adhered to the guidelines outlined in the Preferred Reporting Items for Systematic Reviews and Meta-Analyses (PRISMA) statement (28), and the research protocol was registered on the Prospective Register of Systematic Reviews (PROSPERO) platform (registration number: CRD420251118367).

## 2.1 Literature search strategy

A systematic search for relevant keywords and free-text terms was conducted across eight databases using Boolean logic operators, following the specific search rules of each database. The databases included CNKI (China National Knowledge Infrastructure), Wanfang Data, VIP, PubMed, Embase, the Cochrane Library, Web of Science, and CBM (China Biology Medicine). The search timeframe covered from the inception of each database until May 31, 2025. Search terms included: “sleep”, “sleep disorder”, “insomnia”, “PSQI score”, “exercise”, “single exercise”, “combined exercise”, “aerobic exercise”, “resistance exercise”, “mind-body exercise”, “Qigong exercise”, “yoga”, “tai chi”, “China”, “Chinese”, “college students”, “university students”, and “randomized controlled trial”. This study strictly adhered to the PRISMA guidelines for developing criteria regarding literature searching, inclusion, screening, and exclusion (29).

### 2.2 Literature inclusion and exclusion criteria

The inclusion and exclusion criteria for the literature review were established based on the PICOS (Population; Intervention; Comparison; Outcome; Study design) framework (30). ① Study Design: limited to randomized controlled trials (RCTs), as this methodology is recognized for its rigorous control of confounding variables, thereby enhancing the reliability and comparability of findings. ②Participants: the primary subjects were Chinese middle school, vocational school, or university students diagnosed with sleep disorders. The target population comprised Chinese middle school and university students. The included studies primarily focused on university students, with participant ages ranging from 18 to 29 years. ③ Intervention: the intervention group received an exercise-based intervention, while the control group received no intervention (Non-intervention). ④ Outcome Measure: the primary outcome was the total score of the Pittsburgh Sleep Quality Index (PSQI) before and after the intervention. Exclusion Criteria: ① Studies where the intervention did not involve an exercise modality or where there was no difference in exercise modality between groups; ② Publication types such as theses, conference papers, or reviews; ③ Studies with incomplete, inconsistent, or unreported data, or those using incompatible outcome measures; ④ Duplicate publications; ⑤ Studies involving non-Chinese adolescents, non-sleep disorder patients, or participants outside the specified age range.

### 2.3 Intervention

This study included all randomized controlled trials (RCTs) that compared the effects of physical exercise vs. a control group on sleep disorders in Chinese adolescents. The exercise types reported in these studies were categorized into the following six groups: ① Aerobic Exercise (AE): activities primarily powered by aerobic metabolism, such as jogging, step aerobics, and fitness dancing; ② Resistance Exercise (RE): activities aimed at enhancing muscle strength or endurance by overcoming body weight or external resistance, such as circuit resistance training and strength training; ③ Mind-Body Exercise (ME): activities that integrate consciousness, body, and behavior, such as Fitness Qigong, Tai Chi, and yoga; ④ Combined Aerobic and Resistance Exercise (AE + RE): an intervention combining methods from ① and ②; ① Combined Aerobic and Mind-Body Exercise (AE + ME): an intervention combining methods from ① and ③;⑤ Combined Resistance and Mind-Body Exercise (RE + ME): an intervention combining methods from ② and ③. These interventions aim to improve physical health, enhance sleep self-regulation ability, and promote social interaction, thereby effectively helping adolescents reduce sleep disorders.

### 2.4 Data extraction

Two researchers conducted literature screening and data extraction independently. Any disagreements were resolved through consultation with a third researcher to reach a final decision. The retrieved records were imported into the reference management software NoteExpress. After removing duplicates, the remaining articles were screened by reviewing their titles and abstracts. Subsequently, the full texts of potentially eligible articles were examined to make the final inclusion decision. The following basic information was extracted: first author's name, year of publication, sample sizes of the experimental and control groups, intervention details for the experimental group, outcome assessment tools, and intervention parameters such as session duration, frequency, and total period. This study only counted the participants included in the final reports and those who completed the entire experiment. Therefore, the potential impact of compliance rates and participant withdrawal on the reliability of reported effect sizes was not analyzed.

### 2.5 Risk of bias assessment

The methodological quality of the included studies was assessed using the Cochrane Collaboration's tool for assessing risk of bias (RoB 1.0), as implemented in Review Manager 5.4. This assessment was conducted independently by two researchers. Any disagreements were resolved through discussion with a third researcher to reach a consensus (31). The quality assessment covered seven key domains: (1) Random sequence generation; (2) Allocation concealment; (3) Blinding of participants and personnel; (4) Blinding of outcome assessment; (5) Completeness of outcome data; (6) Selective reporting; (7) Other potential sources of bias. This process ensured a rigorous evaluation of the methodological quality of the included studies.

### 2.6 Statistical synthesis and analysis

Statistical analysis was performed using Stata 18.0 software. The primary outcome was the post-intervention total score on the Pittsburgh Sleep Quality Index (PSQI). Since the PSQI is a universally standardized scale (score range 0–21) across all included studies, the Mean Difference (MD) was planned as the primary effect size metric to preserve clinical interpretability. The change-from-baseline scores were not pooled unless necessary; if used, variance estimation followed the Cochrane Handbook guidelines. This study employed a network meta-analysis approach, which integrates both direct and indirect evidence while considering sample sizes and effect sizes to simultaneously compare the effects of multiple interventions. The ranking of interventions was achieved using the Surface Under the Cumulative Ranking Curve (SUCRA), where a higher SUCRA value indicates a greater probability of being the most effective treatment (32). The network evidence graph was generated using the network package. To ensure the reliability of the results, global and local inconsistency tests were conducted to address heterogeneity among studies. Publication bias was assessed using a funnel plot.

### 2.7 Evidence certainty assessment

The Grading of Recommendations Assessment, Development and Evaluation (GRADE) approach was employed to assess the certainty of evidence for each outcome (33). The rating process was based on the following five domains: risk of bias, inconsistency, indirectness, imprecision, and publication bias. The certainty of evidence was classified into four levels: high, moderate, low, or very low. Evidence from randomized controlled trials started at the high certainty level and was downgraded sequentially according to the assessment of the five domains. The rating process was conducted independently by two reviewers (Li and Gao), and any disagreements were resolved through discussion or adjudication by a third party.

## 3 Results

### 3.1 Literature search results

**Figure 1:**
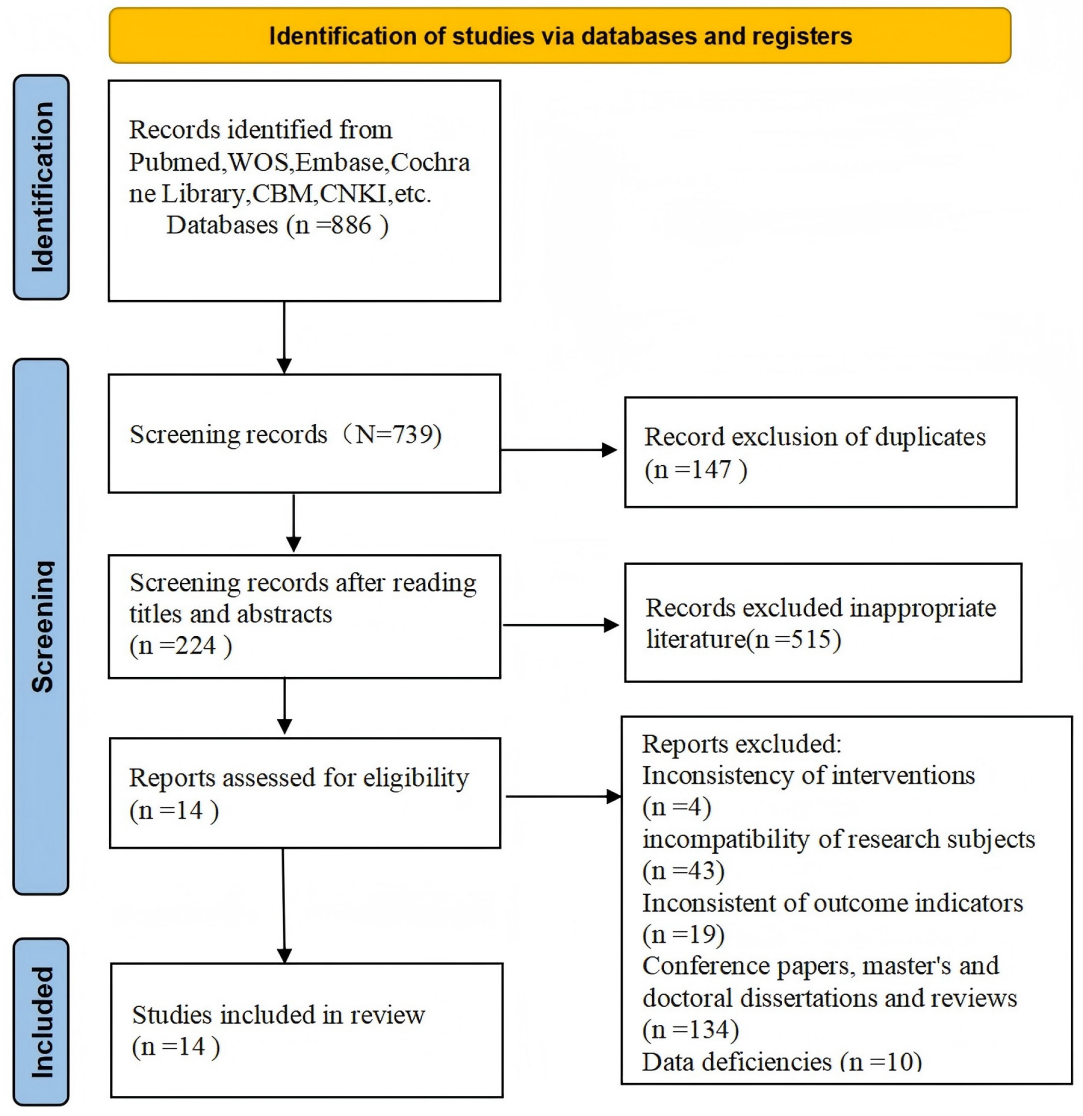
PRISMA flow diagram of the study process.

To ensure the accuracy of the search results, a systematic database search was conducted following the PRISMA guidelines (34). The initial search yielded a total of 886 records. After removing 147 duplicate entries using the NoteExpress reference management software, 739 articles remained for screening. Following a preliminary review of titles and abstracts, 515 articles were excluded, leaving 224 studies for full-text evaluation. Upon further full-text assessment, 210 studies were excluded due to reasons such as missing data, non-matching participant criteria, or the absence of a control group. Ultimately, 14 randomized controlled trials (RCTs) were included in the final analysis. The detailed selection process is illustrated in Figure 1.

### 3.2 Basic characteristics of the included studies

A total of 14 RCTs were included in the final analysis. The overall sample size comprised 1,375 participants, with 718 individuals assigned to intervention groups and 657 to control groups. These studies encompassed six intervention categories: AE, RE, ME, AE + RE, AE + ME, and RE + ME. Specifically, four studies involved AE, one involved RE, six involved ME, two involved AE + RE, three involved AE + ME, and two involved RE + ME. Detailed characteristics of the included studies are presented in Table 1.

**Table 1:** Basic characteristics of the included studies.

References	Sample size (T/C)	Age (T/C)	Exercise type	Duration	Frequency	Period	Outcome indications
Guo et al. (35)	20/20	19.60 ± 0.88	ME	60 min	2/week	4 weeks	PSQI
Luo (36)	157/158	18.58 ± 2.25	ME	30 min	3/week	16 weeks	PSQI
Liu et al. (37)	30/30	20.16 ± 1.37	AE	50 min	7/week	2 weeks	PSQI
Cui and Bai (22)	30/30	18–24	ME	60 min	5/week	8 weeks	PSQI
Zhang et al. (38)	136/136	NR	AE	50 min	3/week	12 weeks	PSQI
Bi et al. (39)	15/15/15/16	21.14 ± 1.58	AE + RE/AE + ME/RE + ME	50 min	3/week	10 weeks	PSQI
Wei et al. (40)	100/100	19–21	ME	90 min	1/week	18 weeks	PSQI
Liu et al. (41)	29/12	18–24	AE	55 min	3/week	8 weeks	PSQI
Xu et al. (24)	43/43	20.73 ± 2.66/20.36 ± 3.09	RE	NR	3/week	12 weeks	PSQI
Yuan et al. (23)	10/8/9/8	18–26	AE + RE/AE + ME/RE + ME	60 min	3/week	8 weeks	PSQI
Zou et al. (42)	19/19	20.47 ± 1.47/20.53 ± 1.93	ME	60 min	4/week	10 weeks	PSQI
Gong et al. (43)	34/36	22.85 ± 1.26	AE + ME	40 min	3/week	8 weeks	PSQI
Sun et al. (44)	18/19	20.9 ± 4.3	ME	60 min	5/week	12 weeks	PSQI
Yin et al. (45)	30/30	18–22	AE	45–60 min	3/week	4 weeks	PSQI

AE, aerobic exercise; RE, resistance exercise; ME, mind-body exercise; NR, not reported; PSQI, Pittsburgh Sleep Quality Index.

As shown in Table 1, the sample sizes in some studies were relatively small, such as in the study by Yuan et al. (23). Smaller sample sizes may affect the stability and reliability of the findings. However, this factor was fully considered in the subsequent analysis, and multiple methods were employed for comprehensive evaluation to minimize its potential impact on the study results.

### 3.3 Risk of bias assessment results

**Figure 2:**
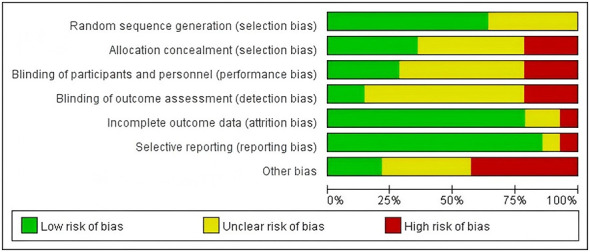
Bias risk diagram.

**Figure 3:**
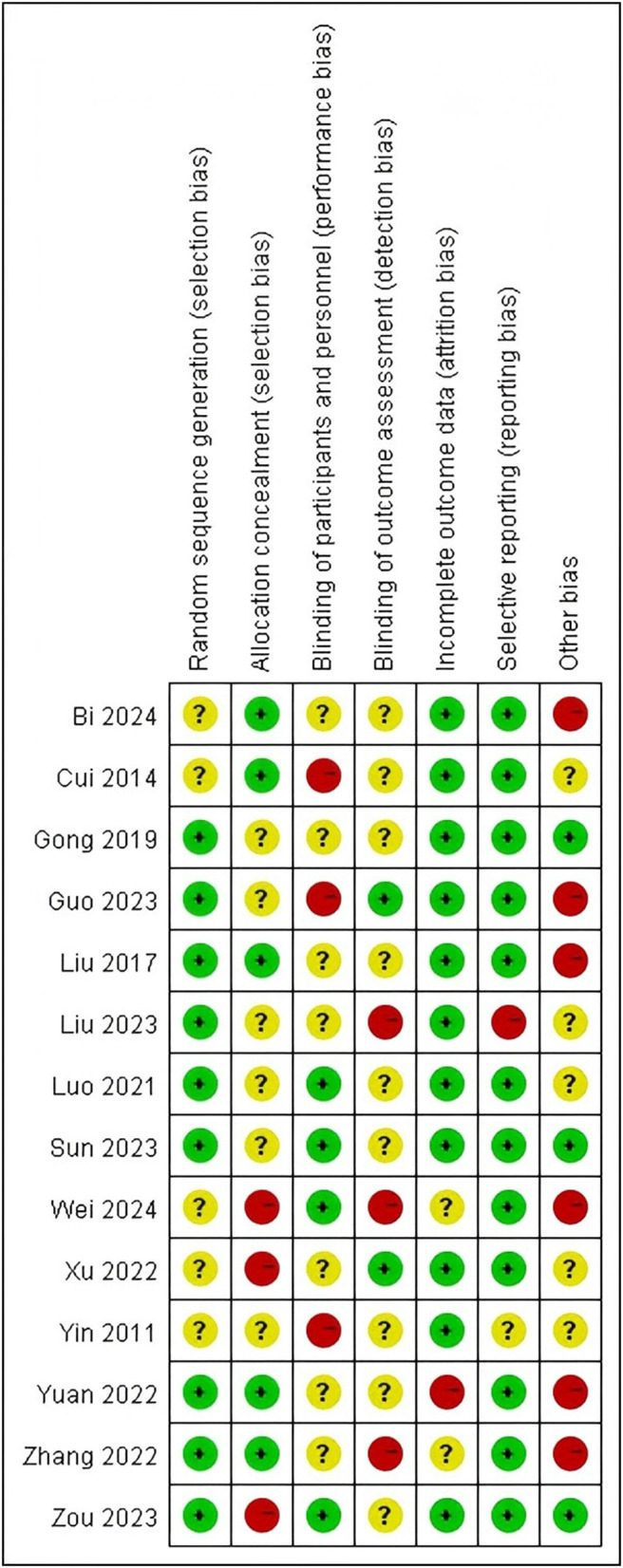
Risk of bias summary.

The risk of bias analysis, conducted using the Cochrane Risk of Bias tool (RoB 1.0), indicated that the primary source of bias in the included studies was related to the randomization process. According to the RoB 1.0 criteria, a study is classified as having an overall low risk of bias if all items are rated “low risk”. An overall moderate risk of bias is assigned if one or two items are rated as “high risk” or “some concerns”. An overall high risk of bias is assigned if more than two items are rated as “high risk” or “some concerns.” The detailed results of the risk of bias assessment are presented in Figures 2, 3.

### 3.4 Inconsistency tests

Inconsistency refers to the discrepancy between direct and indirect evidence in a network meta-analysis, which may affect the reliability of the results. When conducting a network meta-analysis, if closed loops exist within the network of interventions, both global and local inconsistency tests are required to assess the consistency between direct and indirect comparisons. If no closed loops are present, inconsistency tests are unnecessary. Global inconsistency was assessed only for the “Exercise Type” network, as it was the only network containing closed loops. The *P*-value for the global inconsistency test was 0.9012, indicating good consistency (exceeding the 0.05 threshold). Therefore, a consistency model was adopted for further analysis (27). In the network structure diagram, each node represents an intervention. The connections between nodes represent the effects between two interventions. Node-splitting is a method that separates the data for each direct comparison from all other data (46). Local inconsistency was tested using the node-splitting method. The results (*P* > 0.05) indicated good local consistency and no heterogeneity among the different comparisons. Consequently, the consistency model was used for analysis in this study. However, no closed loops were formed among the variables of duration, frequency and period networks; therefore, consistency tests were not required for these parameters.

### 3.5 Network meta-analysis results

#### 3.5.1 Network plot

**Figure 4:**
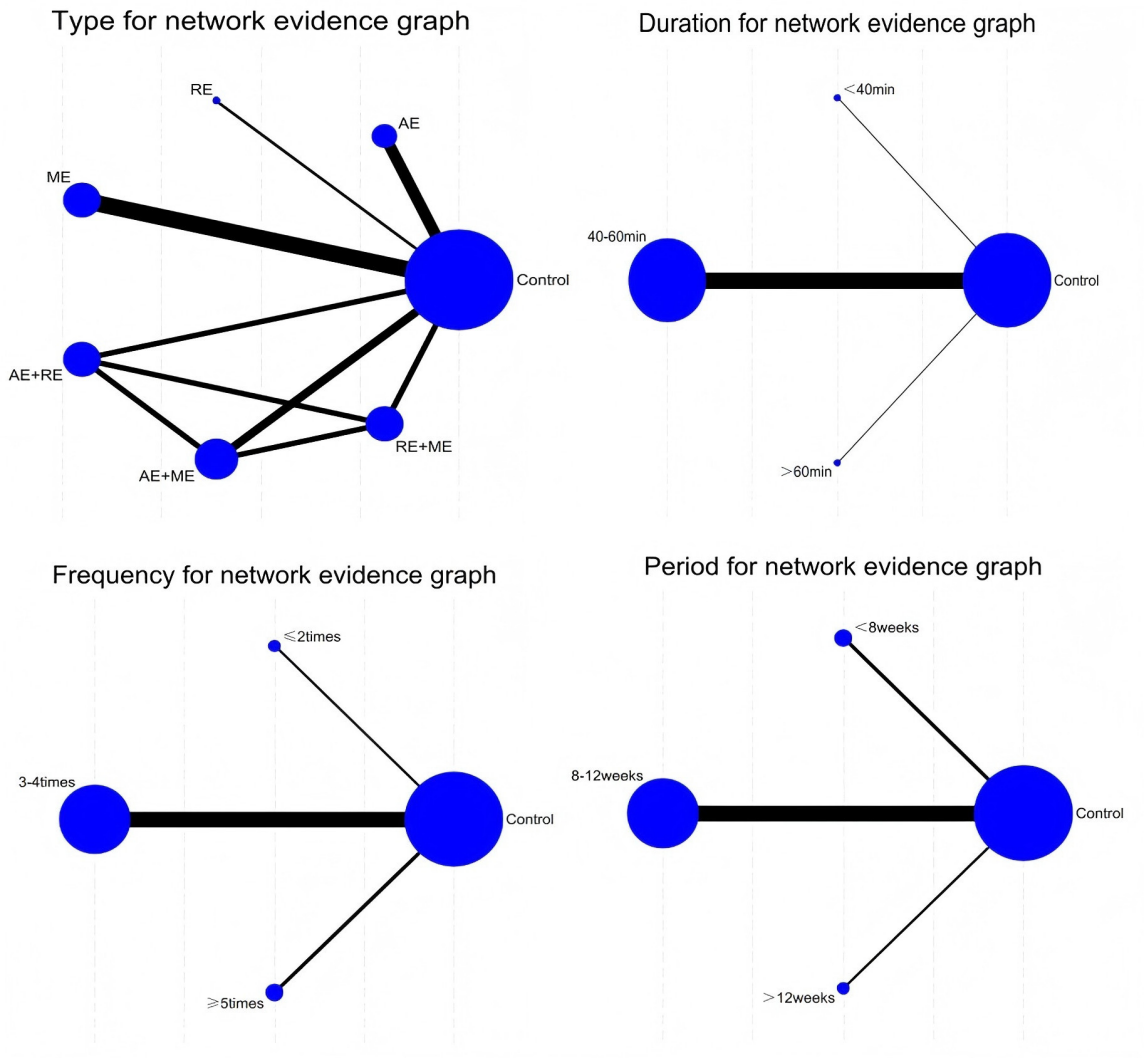
Network evidence diagram for type, duration, frequency and period.

This study synthesized 14 high-quality randomized controlled trials (RCTs) to scientifically evaluate the therapeutic effects of various exercise modalities, intervention duration, frequencies, and total periods in treating adolescent sleep disorders. In the network diagram, each node represents a distinct intervention, with its size proportional to the corresponding intervention's sample size. The thickness of the lines connecting the nodes indicates the number of studies directly comparing the linked interventions. Direct comparisons refer to head-to-head contrasts between two interventions. Closed loops within the network signify the presence of both direct and indirect comparisons for a set of interventions. The specific network graph is presented in Figure 4 (utilizing the lower limit when ranges are specified).

#### 3.5.2 Pairwise meta-analysis results

A network meta-analysis was conducted on the included studies. Mean differences (MD) were used for pairwise comparisons between interventions, and 95% confidence intervals (CI) were applied to ensure the precision and reliability of the results at the 95% confidence level. As shown in Table 2, based on the pairwise comparison results, the intervention effects of aerobic exercise [MD = 1.94, 95% CI (0.15, 3.74)], mind-body exercise [MD = 3.34, 95% CI (1.89, 4.79)], combined aerobic and resistance exercise [MD = 3.34, 95% CI (0.55, 6.13)], and combined aerobic and mind-body exercise [MD = 4.14, 95% CI (1.93, 6.36)] were all significantly superior to those of the non-intervention control group. Furthermore, the intervention effect of a session duration of 40–60 min [MD = 3.18, 95% CI (2.20, 4.15)] was significantly different from that of the control group, whereas session durations of <40 min and >60 min showed no statistically significant differences. Intervention frequencies of 3–4 times per week [MD = 2.81, 95% CI (1.80, 3.83)] and ≥5 times per week [MD = 4.05, 95% CI (2.09, 6.01)] both produced effects significantly superior to those of the control group, while a frequency of ≤ 2 times per week did not show a statistically significant difference. Regarding the total intervention period, durations of <8 weeks [MD = 2.34, 95% CI (0.32, 4.37)] and 8–12 weeks [MD = 3.24, 95% CI (2.19, 4.30)] both had intervention effects significantly better than those of the control group, whereas a duration of >12 weeks showed no statistically significant difference.

**Table 2:** League table of comparative effectiveness results for type, duration, frequency and period.

Comparison	Control						
AE	**1.94 (0.15, 3.74)**	AE					
RE	1.40 (−2.12, 4.92)	0.54 (−3.41, 4.50)	RE				
ME	**3.34 (1.89, 4.79)**	−1.39 (−3.70, 0.92)	−1.94 (−5.74, 1.87)	ME			
AE + RE	**3.34 (0.55, 6.13)**	−1.40 (−4.72, 1.92)	−1.94 (−6.43, 2.55)	−0.00 (−3.15, 3.14)	AE + RE		
AE + ME	**4.14 (1.93, 6.36)**	−2.20 (−5.05, 0.66)	−2.74 (−6.90, 1.42)	−0.80 (−3.45, 1.84)	−0.80 (−3.50, 1.90)	AE + ME	
RE + ME	2.24 (−0.47, 4.95)	−0.30 (−3.55, 2.95)	−0.84 (−5.29, 3.60)	1.09 (−1.98, 4.17)	1.10 (−1.76, 3.96)	1.90 (−0.72, 4.51)	RE + ME
Comparison	Control						
<40 min	**2.65 (−0.81, 6.11)**	<40 min					
40–60 min	**3.18 (2.20, 4.15)**	−0.53 (−4.12, 3.07)	40–60 min				
>60 min	0.97 (−2.60, 4.54)	1.68 (−3.29, 6.65)	2.21 (−1.50, 5.91)	>60 min			
Comparison	Control						
≤ 2 times	**1.62 (−0.80, 4.05)**	≤ 2 times					
3–4 times	**2.81 (1.80, 3.83)**	−1.19 (−3.82, 1.44)	3–4 times				
≥5 times	**4.05 (2.09, 6.01)**	−2.43 (−5.54, 0.69)	−1.24 (−3.44, 0.97)	≥5 times			
Comparison	Control						
<8 weeks	**2.34 (0.32, 4.37)**	<8 weeks					
8–12 weeks	**3.24 (2.19, 4.30)**	0.90 (−1.38, 3.18)	8–12 weeks				
>12 weeks	**1.84 (−0.62, 4.29)**	−0.51 (−3.69, 2.67)	−1.41 (−4.08, 1.27)	>12 weeks			

Bold numbers indicate statistical significance; The numbers in the table represent the results of pairwise comparisons.

#### 3.5.3 Ranking of the effectiveness of each element in the exercise prescription

**Figure 5:**
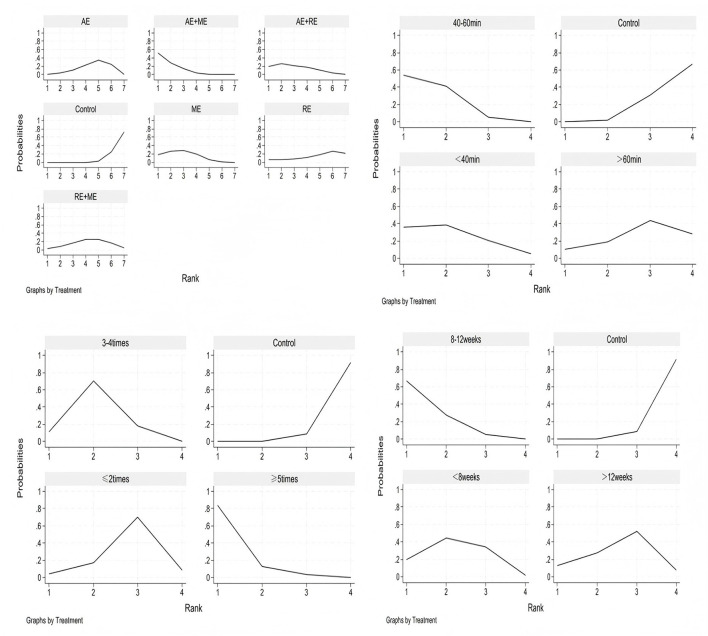
Results of the SUCRA. Type, Duration, Frequency, Period.

One of the key features and primary strengths of network meta-analysis is its ability to rank the comparative effectiveness of interventions. This is commonly achieved using the SUCRA (Surface Under the Cumulative Ranking Curve) method, which calculates the cumulative probability for each intervention to be the best, second best, and so on, based on its relative effects in all pairwise comparisons, thereby determining its ranking probability among all interventions. Figure 5 presents the SUCRA values; a higher SUCRA value indicates a greater probability of superior efficacy. As shown in Table 3 and Figure 5, the SUCRA ranking for various exercise interventions on adolescent sleep disorders is as follows: AE + ME (SUCRA = 87.1%) >ME (SUCRA = 71.3%) >AE + RE (SUCRA = 68.5%) >RE + ME (SUCRA = 44.7%) >AE (SUCRA = 40.0%) >RE (SUCRA = 33.4%). The ranking for different intervention session duration is: 40–60 min (SUCRA = 82.9%) > <40 min (SUCRA = 68.7%) >60 min (SUCRA = 36.7%). The ranking for different intervention frequencies is: ≥5 times/week (SUCRA = 93.6%) >3–4 times/week (SUCRA = 64.5%) > ≤ 2 times/week (SUCRA = 39.0%). The ranking for different total intervention periods is: 8–12 weeks (SUCRA = 87.3%) > <8 weeks (SUCRA = 61.1%) >12 weeks (SUCRA = 48.7%). These ranking results are clinically meaningful. They can guide future research by highlighting the most promising intervention parameters, allowing researchers to prioritize investigating the highest-ranked combinations to optimize treatment strategies for adolescent sleep disorders.

**Table 3:** SUCRA values of intervention effects for type, duration, frequency and period.

Type	SUCRA	Duration	SUCRA	Frequency	SUCRA	Period	SUCRA
Control	5.0	Control	11.6	Control	2.9	Control	2.9
AE	40.0	<40 min	68.7	≤ 2 times	39.0	<8 weeks	61.1
RE	33.4	40–60 min	82.9	3–4 times	64.5	8–12 weeks	87.3
ME	71.3	>60 min	36.7	≥5 times	93.6	>12 weeks	48.7
AE + RE	68.5						
AE + ME	87.1						
RE + ME	44.7						

#### 3.5.4 Publication bias test

**Figure 6:**
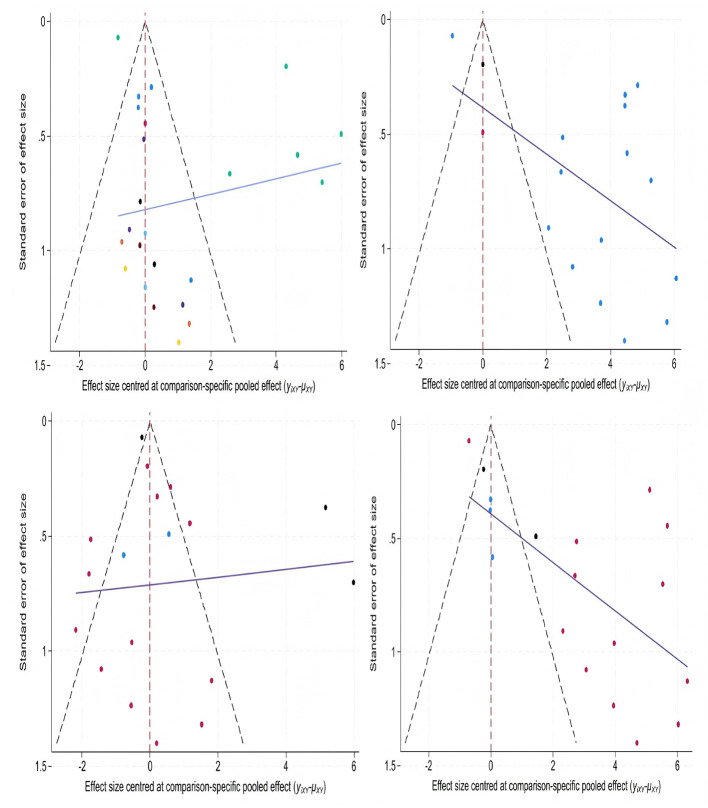
Funnel plot of the effect of each intervention. Type, Duration, Frequency, Period.

Publication bias was assessed using a funnel plot. The horizontal axis represents the effect size, the vertical axis represents the standard error, and the dots represent the individual studies. A comparison-adjusted funnel plot was generated for the exercise type network to account for multiple comparisons. The interventions were ordered according to their chronological year of introduction into clinical practice or, alternatively, by SUCRA ranking to visually assess small-study effects. A symmetrical distribution of dots indicates a low risk of publication bias. As shown in Figure 6, the funnel plots for each intervention network exhibited a generally symmetrical inverted funnel-shaped distribution. Specifically, for exercise type, most study points were concentrated near the top center of the funnel plot, indicating high precision. The funnel plots for intervention duration, frequency, and total period also showed good symmetry, with no obvious small-study effects observed. The result of Egger's test (*p* = 0.052), although close to the threshold, did not reach statistical significance, suggesting potential publication bias and warranting cautious interpretation. Taken together, the funnel plots and Egger's test results indicate that the overall risk of publication bias in this study is low, although potential bias cannot be ruled out for certain comparisons.

#### 3.5.5 Sensitivity analysis

**Figure 7:**
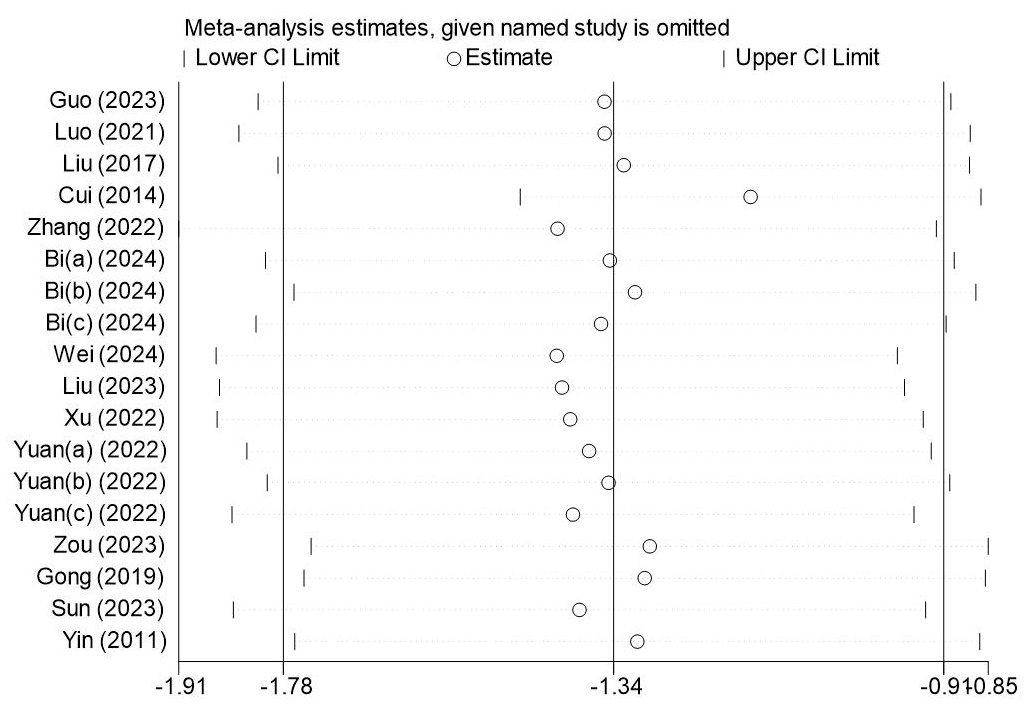
Sensitivity analysis. The X-axis label is effect size (95% CI), and the Y-axis label is study omitted.

Figure 7 presents the pooled effect estimates after sequentially excluding individual studies using the leave-one-out method for sensitivity analysis. The central circle represents the pooled effect size, and the extending lines indicate the confidence interval. The stability of the findings is reflected in the absence of significant changes in the overall effect estimate. The leave-one-out sensitivity analysis involved iteratively removing one study at a time to assess its impact on the overall effect size. As shown in Figure 7, the effect estimates fluctuated within a narrow range of −1.91 to −0.85 following this procedure. This range indicates that the removal of any single study did not substantially alter the pooled effect estimate, highlighting the robustness of the analysis. This finding demonstrates that the synthesized effect size is not unduly influenced by any specific included study, thereby enhancing the overall reliability of the results.

### 3.6 Evidence certainty assessment results

The Grading of Recommendations Assessment, Development and Evaluation (GRADE) approach was used to assess the certainty of evidence for the main findings of this network meta-analysis. The assessment was based on five downgrading domains: risk of bias, inconsistency, indirectness, imprecision, and publication bias. The primary outcome measure was the change in total PSQI score post-intervention. Given the heterogeneity of the evidence base across different comparisons in the network meta-analysis, we conducted graded assessments for the main types of exercise and for the exercise prescription parameters that showed statistical significance. Detailed results are presented in Table 4.

**Table 4:** GRADE evidence profile.

Outcome indicator	Number of studies	Evidence quality assessment					Effect size	Evidence quality
		Risk of bias	Inconsistency	Indirectness	Imprecision	Publication bias		
ME	6	Downgraded (−1)	Not downgraded	Not downgraded	Not downgraded	Not downgraded	3.34 (1.89, 4.79)	Moderate
AE	4	Downgraded (−1)	Not downgraded	Not downgraded	Not downgraded	Not downgraded	1.94 (0.15, 3.74)	Moderate
AE + RE	2	Downgraded (−1)	Not downgraded	Not downgraded	Downgraded (−1)	Not downgraded	3.34 (0.55, 6.13)	Low
AE + ME	3	Downgraded (−1)	Not downgraded	Not downgraded	Downgraded (−1)	Not downgraded	4.14 (1.93, 6.36)	Low
40–60 min	11	Downgraded (−1)	Not downgraded	Not downgraded	Not downgraded	Not downgraded	3.18 (2.20, 4.15)	Moderate
≥5 times/week	3	Downgraded (−1)	Not downgraded	Not downgraded	Not downgraded	Not downgraded	4.05 (2.09, 6.01)	Moderate
3–4 times/week	9	Downgraded (−1)	Downgraded (−1)	Not downgraded	Not downgraded	Not downgraded	2.81 (1.80, 3.83)	Low
8–12 weeks	9	Downgraded (−1)	Downgraded (−1)	Not downgraded	Not downgraded	Not downgraded	3.44 (2.38, 4.50)	Low
<8 weeks	3	Downgraded (−1)	Not downgraded	Not downgraded	Downgraded (−1)	Not downgraded	2.34 (0.42, 4.27)	Moderate

#### 3.6.1 Certainty of evidence for exercise types

For the comparison of mind-body exercise vs. a non-intervention control, the certainty of evidence was moderate. This rating was based on six randomized controlled trials. The evidence was downgraded by one level due to risk of bias: although the overall quality of the included studies was acceptable, most studies had a “high risk of bias” or “some concerns” regarding “allocation concealment” and “blinding of participants and personnel.” This limitation is inherent to non-pharmacological interventions, where blinding of instructors and participants is difficult to achieve. No additional downgrading was applied for inconsistency, indirectness, imprecision, or publication bias. For the comparison of aerobic exercise vs. the control, the certainty of evidence was also moderate, with the same rationale for downgrading as for mind-body exercise.

For combined aerobic and resistance exercise and combined aerobic and mind-body exercise, although pairwise comparisons showed significant effects superior to the control group, the certainty of evidence was downgraded to low. This downgrade was due to the small number of studies providing direct comparisons (two studies for AE + RE and three studies for AE + ME), the relatively small sample sizes in some studies, and the wide confidence intervals around the effect estimates. This indicates considerable uncertainty regarding the effect estimates for these combined interventions, and future research is likely to change the confidence in these estimates. Resistance exercise and combined resistance and mind-body exercise were rated as having very low certainty evidence due to the very limited number of studies (one and two studies, respectively) and the imprecise effect estimates.

#### 3.6.2 Certainty of evidence for exercise prescription parameters

Regarding exercise parameters, based on the pairwise comparison results (see Table 2), the parameters that showed statistical significance were session duration of 40–60 min [MD = 3.18, 95% CI (2.20, 4.15)], frequencies of 3–4 times per week [MD = 2.81, 95% CI (1.80, 3.83)] and ≥5 times per week [MD = 4.05, 95% CI (2.09, 6.01)], and total intervention periods of <8 weeks [MD = 2.34, 95% CI (0.32, 4.37)] and 8–12 weeks [MD = 3.24, 95% CI (2.19, 4.30)].

Among these, the comparisons for session duration of 40–60 min, frequency of ≥5 times per week, and total period of <8 weeks vs. the control were all rated as having moderate certainty evidence. The primary reason for downgrading was risk of bias. Additionally, for the parameters of frequency ≥5 times per week and total period <8 weeks, the small number of included studies increased the imprecision of the results; therefore, downgrading was applied for imprecision.

For the parameters of frequency of 3–4 times per week and total period of 8–12 weeks, although the effects were significant, the certainty of evidence was rated as low due to some clinical heterogeneity in the specific intervention frequencies and durations across the included studies, as well as concerns about risk of bias in some studies.

#### 3.6.3 Overall summary of evidence

In summary, in the current body of evidence, mind-body exercise, aerobic exercise, and the exercise prescription parameters of a 40–60 min session duration, a frequency of ≥5 times per week, and a total intervention period of <8 weeks are supported by relatively higher certainty evidence (moderate). In contrast, the evidence base for most combined exercise modalities and other parameter levels is weaker (low or very low). This suggests that while SUCRA rankings provide a reference for comparative effectiveness, readers should pay particular attention to the underlying certainty of evidence when interpreting these rankings. Clinical decisions should prioritize intervention regimens supported by moderate-certainty evidence.

## 4 Discussion

This study employed a network meta-analysis to systematically synthesize evidence from randomized controlled trials on exercise interventions for sleep disorders in Chinese adolescents. It comprehensively evaluated the effects of both single and combined exercise modalities, along with various exercise parameters including session duration, frequency, and total intervention period. The findings provide novel insights for developing optimal exercise prescription frameworks for individuals with sleep disorders and offer more actionable and refined references for future clinical practice and scientific research in this field.

The validity of network meta-analysis relies on the transitivity assumption. We assessed this by examining the distribution of potential effect modifiers across the included comparisons. The populations were all Chinese students with sleep disorders, and the control conditions were consistently non-intervention groups. Although there were variations in specific exercise parameters, the interventions were broadly comparable in terms of intensity and setting. Therefore, we considered the transitivity assumption to be plausible for this analysis.

The network meta-analysis results indicate that, based on SUCRA rankings (Table 3), the combination of aerobic and mind-body exercise (AE + ME) demonstrated the highest probability of being the most effective intervention (SUCRA = 87.1%), followed by mind-body exercise alone (ME, SUCRA = 71.3%) and combined aerobic and resistance exercise (AE + RE, SUCRA = 68.5%). However, pairwise comparison results (Table 2) showed that ME, AE, AE + RE, and AE + ME all had significant effects compared to the control group, with no statistically significant differences detected among these active interventions. The optimal session duration was 40–60 min [MD = 3.18, 95% CI (2.20, 4.15)], with a frequency of ≥5 times per week [MD = 4.05, 95% CI (2.09, 6.01)], and the ideal total intervention period was less than 8 weeks [MD = 2.34, 95% CI (0.32, 4.37)]. The GRADE assessment (Table 4) suggests that the certainty of evidence supporting ME, AE, and the optimal parameters (40–60 min, ≥5 times/week, <8 weeks) is moderate, implying that future research is likely to have an important impact on our confidence in these effect estimates.

This study found that mind-body exercises, represented by practices such as Tai Chi, Baduanjin, and yoga, demonstrate significant intervention effects in improving sleep disorders among adolescents [MD = 3.34, 95% CI (1.89, 4.79)]. Consistent with previous research on sleep improvement, exercises emphasizing the integration of mind and body, like Tai Chi, Qigong, and yoga, are common and effective interventions for enhancing sleep quality (19). Notably, although AE + ME had the highest SUCRA value, its pairwise comparison effect [MD = 4.14, 95% CI (1.93, 6.36)] was not statistically significantly different from ME alone, and the evidence quality for AE + ME was rated as low due to the limited number of studies (three studies) and wide confidence intervals (Table 4). The superior performance of ME may be particularly meaningful for adolescents, who are navigating a critical period marked by high psychological stress, emotional volatility, and social role transitions. ME typically involves slow, controlled physical movements, breath-focused meditation, and mindful awareness. This combination effectively regulates autonomic nervous system balance by enhancing parasympathetic activity and reducing sympathetic dominance. Studies indicate that regular practice of Tai Chi or yoga can significantly lower resting heart rate, blood pressure, and cortisol levels—physiological changes highly consistent with the state of sleep readiness (19). Furthermore, sleep disorders are often associated with pre-sleep hyperarousal and physiological activation. The mind-body relaxation skills cultivated through regular ME practice can be directly transferred to the bedtime period, helping to reduce sleep onset latency (35). Adolescence is a stage of identity formation and intense peer pressure. ME emphasizes non-competitiveness, self-acceptance, and internal focus. Forms such as Tai Chi, Baduanjin, and yoga feature gentle movements, possess a long developmental history in China, and offer rich content, potentially making them more acceptable and sustainable for Chinese adolescents. This may contribute to reducing sleep latency, extending total sleep time, and thereby improving sleep efficiency (47). Therefore, the pronounced intervention effect of mind-body exercise on adolescent sleep disorders is likely attributable to its advantages—characterized by slow, gentle movements, higher acceptance, and the promotion of physical and mental relaxation—compared to other forms of exercise.

Aerobic exercise, as a common exercise modality, also demonstrated statistically significant improvement compared to the control group [MD = 1.94, 95% CI (0.15, 3.74)], confirming the universal benefits of exercise intervention. While the effect of AE was less pronounced than that of ME and certain combined regimens, its strong foundation for use and ease of implementation make it a fundamental and indispensable option. Aerobic exercise can improve sleep quality by influencing physiological processes such as thermoregulation, endocrine function, autonomic nervous system regulation, and metabolic activity (48, 49). Erlacher et al. (48); Katofsky et al. (50) have confirmed that jogging can effectively enhance sleep quality in university students, which aligns with the findings of the present research. It is noteworthy that RE intervention did not demonstrate a superior advantage in improving sleep disorders among adolescents [MD = 1.40, 95% CI (−2.12, 4.92), not statistically significant]. A plausible explanation is that resistance exercise, particularly moderate- to high-intensity training, induces an acute stress response affecting the hypothalamic-pituitary-adrenal (HPA) axis and the hypothalamic-pituitary-gonadal (HPG) axis, characterized by a transient increase in stress hormones such as cortisol and catecholamines (51, 52). In adults, this acute stress is typically followed by a recovery period with compensatory relaxation and hormonal normalization, which may facilitate subsequent deep sleep. However, adolescents are in a developmental stage marked by highly active yet not fully matured HPA and HPG axes, with fine regulatory mechanisms still developing. Research indicates heightened sensitivity of the HPA axis to stimuli during puberty, and post-stress recovery capacity may be lower than in adults (53). This physiological profile runs counter to the intervention goal of promoting sleep through relaxation. In contrast, ME explicitly targets stress reduction. While moderate-intensity AE also activates the HPA axis, its response pattern is likely more moderate, and its subsequent core body temperature-lowering effect is more conducive to sleep initiation.

The performance of combined exercise regimens warrants further discussion. In this study, both the AE + RE and AE + ME combinations achieved higher SUCRA rankings than single AE or RE interventions, with AE + ME showing the highest ranking (SUCRA = 87.1%). This suggests that combining two exercise modalities with distinct physiological mechanisms may yield complementary or synergistic effects. Neuroimaging evidence substantiates that aerobic exercise can trigger endorphin release, enhance mood, and reduce stress (54). Resistance training can significantly increase muscle mass, thereby elevating resting metabolic rate, and is also an effective means of improving insulin sensitivity and glucose metabolism (55). A randomized controlled trial in adults found that a combined AE and RE program not only improved subjective sleep quality but also more effectively reduced systemic inflammatory markers (56). Given that adolescent sleep disorders are often associated with chronic low-grade inflammation and dysregulated stress hormones, the AE + RE regimen may produce superior sleep-promoting effects through this combined endocrine and immunomodulatory action. The significant intervention effect of the AE + ME regimen on adolescent sleep disorders may be attributed to the fact that aerobic exercise safely elevates sympathetic nervous system activity and promotes cardiovascular adaptation. Meanwhile, mind-body exercise, through slow movements, focused breathing, and mindfulness meditation, potently activates the parasympathetic nervous system, modulates the sympathetic-vagal balance, and induces a deep relaxation response (57). This is particularly well-suited for alleviating the state of sympathetic overexcitation and pre-sleep tension in adolescents caused by academic pressure and social anxiety. However, the evidence quality for these combined regimens was rated as low (Table 4), indicating considerable uncertainty. Therefore, despite their high SUCRA rankings, these findings should be interpreted with caution. Future research should delve deeper into optimizing the mode, sequence, duration, and dose ratios of combined exercise interventions, moving beyond simplistic combination.

This study indicates that an exercise session duration of 40–60 min is most effective for improving sleep disorders in adolescents, as shorter (<40 min) and longer (>60 min) durations showed no statistically significant differences compared to the control group (Table 2). Consistent with prior research, a specific study noted that adolescents who accumulate between 30 and 60 min of moderate-to-vigorous physical activity daily demonstrate significantly better subjective sleep quality and objectively measured sleep efficiency compared to their less active peers (58). This effect may stem from the thermogenic process during exercise. Muscle activity generates heat, causing a significant rise in core body temperature. Subsequently, the body enhances heat dissipation through mechanisms such as vasodilation and sweating. Within 1–1.5 h after exercise cessation, core temperature drops to a level below the pre-exercise baseline. This post-exercise decline in body temperature coincides with the body's natural pre-sleep thermoregulatory rhythm and is widely recognized as a key physiological signal promoting sleep onset (59). A 40–60 min session of moderate-intensity exercise is sufficient to induce a pronounced and sustained body temperature fluctuation cycle. Sessions that are too short may not trigger a robust enough thermoregulatory response, while sessions that are excessively long may lead to profuse sweating, fluid imbalance, and prolonged elevated body temperature. This can not only delay the cooling process but may also cause dehydration and discomfort, thereby hindering a smooth transition to sleep. Therefore, an intervention duration of 40–60 min demonstrates the most significant effect in improving sleep quality among adolescents.

The study demonstrates that a high intervention frequency of ≥5 sessions per week is most effective for improving sleep disorders in adolescents [MD = 4.05, 95% CI (2.09, 6.01)], with moderate certainty evidence (Table 4). This finding aligns with existing evidence indicating that adolescents with higher daily physical activity levels, which typically implies higher activity frequency, exhibit better subjective and objective sleep quality (58). Sleep is a daily physiological process, and its regulation also requires consistent, daily intervention input. High-frequency exercise can more stably modulate circadian rhythm signals and provide a more continuous outlet for emotional relief and stress release, thereby exerting both cumulative and immediate positive effects on sleep (60). Therefore, exercising ≥5 times per week helps deeply integrate the behavior into the daily life rhythm, forming a strong behavioral habit. In contrast, a lower frequency may be insufficient to generate a robust and sustained physiological and psychological impact capable of counteracting the daily stressors that contribute to sleep disorders.

The study confirms that an intervention period of less than 8 weeks is effective for enhancing sleep quality in adolescents [MD = 2.34, 95% CI (0.32, 4.37)], with moderate certainty evidence. It is worth noting that the 8–12 week period also showed a significant effect [MD = 3.24, 95% CI (2.19, 4.30)], but the certainty of evidence for this parameter was rated as low due to clinical heterogeneity across studies (Table 4). Previous research has found that sleep improvements likely stem, in part, from the cumulative effect of acute or sub-acute benefits derived from exercise, which can manifest within several weeks of consistent practice (61). However, this does not imply that long-term exercise is unimportant. The significance of a short-term, intensive intervention lies in its ability to quickly break the cycle of worsening sleep and establish initial success experiences and exercise habits. Building upon this foundation, designing strategies to maintain the exercise behavior for long-term consolidation of sleep benefits becomes the key focus of subsequent practice. The results of this study support setting the initial intervention goal as a 6 to 8-week intensive phase with high adherence.

Although this study conducted a meta-analysis of randomized controlled trials to explore the impact of exercise interventions on adolescent sleep disorders, several limitations warrant consideration. First, the number of included studies is limited, and the participants were exclusively Chinese adolescents. Factors such as cultural background, educational systems, and lifestyle may significantly influence both the implementation of exercise programs and their effects on sleep, thereby limiting the generalizability of the conclusions to other populations. More importantly, the GRADE assessment (Table 4) revealed that, with the exception of mind-body exercise, aerobic exercise, and specific exercise parameters (40–60 min, ≥5 times/week, <8 weeks) which were supported by moderate certainty evidence, the certainty of evidence for most combined exercise interventions (AE + RE, AE + ME) and other parameter levels (e.g., 3–4 times/week, 8–12 weeks) was low. Resistance exercise and RE + ME were rated as having very low certainty evidence. This suggests that although the SUCRA rankings provide information on the relative effectiveness of interventions, our confidence in these rankings is limited, particularly for lower-ranked interventions (such as resistance exercise) and parameter levels based on a small number of studies or with significant heterogeneity. Therefore, the conclusions of this study should be regarded as preliminary recommendations based on the best available evidence, rather than definitive conclusions.

Second, all studies uniformly used the total PSQI score as the primary outcome. While the PSQI is a reliable subjective self-report measure, it cannot replace objective sleep monitoring. Exercise may differentially affect subjective sleep perception and objective sleep architecture; future research requires comprehensive assessments combining both subjective and objective measures. Furthermore, this study did not perform subgroup analyses or meta-regression based on factors such as gender, specific age groups, or subtypes of sleep disorders, which further constrains the generalizability of the findings. Subsequent research should account for these factors and develop more targeted intervention programs based on subtype classifications. Additionally, comparisons between some interventions relied on indirect evidence. Although both global and local inconsistency tests yielded satisfactory results, the validity of these findings may still be impacted.

## 5 Conclusion

This study included 14 studies and employed a network meta-analysis to evaluate the effectiveness of single vs. combined exercise modalities in intervening sleep disorders among Chinese adolescents, while also exploring the effects of different exercise prescription variables, such as session duration, frequency, and total period. Although the SUCRA rankings indicated that AE + ME (87.1%) and ME (71.3%) had high probabilities of being the most effective interventions, the GRADE assessment revealed that, with the exception of ME and AE which were supported by moderate-certainty evidence, the certainty of evidence for most combined exercise modalities (including AE + ME) was low or very low. Pairwise comparisons showed that mind-body exercise [MD = 3.34, 95% CI (1.89, 4.79)], aerobic exercise [MD = 1.94, 95% CI (0.15, 3.74)], AE + RE [MD = 3.34, 95% CI (0.55, 6.13)], and AE + ME [MD = 4.14, 95% CI (1.93, 6.36)] all had significant effects compared to the control group, with no statistically significant differences detected among these active interventions. Therefore, based on the current moderate-certainty evidence, ME (40–60 min, ≥5 times/week, <8 weeks) may represent the most reliable regimen for improving sleep disorders in Chinese adolescents.

However, due to the low or very low certainty of evidence for most combined exercise interventions (AE + RE and AE + ME were rated as low certainty; RE and RE + ME as very low certainty) and the weak evidence base for certain exercise parameters (e.g., 3–4 times/week and 8–12 weeks were rated as low certainty), these conclusions should be interpreted with caution. Future large-sample, high-quality, long-term randomized controlled trials are warranted to validate and optimize these exercise prescription parameters. Specifically, the reviewed studies did not account for individual preferences, exercise tolerance, specific characteristics of the sleep problems, or potential gender differences in the effects of exercise on sleep. Further research is urgently needed to strengthen the evidence base, address the existing limitations identified in this review, and thereby enhance the comprehensiveness and credibility of the evidence.

## Data Availability

The original contributions presented in the study are included in the article/supplementary material, further inquiries can be directed to the corresponding author.
